# Overuse of Non-caloric Sweeteners in Foods and Beverages in Chile: A Threat to Consumers' Free Choice?

**DOI:** 10.3389/fnut.2020.00068

**Published:** 2020-06-17

**Authors:** Verónica Sambra, Sandra López-Arana, Paola Cáceres, Karen Abrigo, Javiera Collinao, Alexandra Espinoza, Sabrina Valenzuela, Bielka Carvajal, Gabriel Prado, Rebeca Peralta, Martin Gotteland

**Affiliations:** ^1^Department of Nutrition, Faculty of Medicine, University of Chile, Santiago, Chile; ^2^Faculty of Medicine, School of Nutrition and Dietetics, University of Chile, Santiago, Chile; ^3^Department of Women and Newborn's Health Promotion, Faculty of Medicine, University of Chile, Santiago, Chile; ^4^Human Nutrition Unit, Institute of Nutrition and Food Technology (INTA), University of Chile, Santiago, Chile

**Keywords:** non-caloric sweeteners, acceptable daily intake, food labeling, obesity, children, sucralose, steviol glycosides, acesulfame-K

## Abstract

The prevalence of obesity among Chilean adults and children is one of the highest worldwide. To fight the constant increase of non-communicable diseases and the growing sales of sugar-sweetened beverages, the Chilean government recently enacted a new Law of Food Labeling and Advertising imposing the application of front-of-package warning labels in foodstuffs whose composition exceeds limits for critical nutrients including sugar. Accordingly, food companies have been reformulating their products, incorporating non-caloric sweeteners (NCSs) in partial or total replacement of sucrose. The number of NCS-containing foods and beverages, therefore, has been increasing in the last years. This study aims to identify the NCS-containing products from different food/beverage categories currently available on the Chilean market. Nineteen supermarkets and 13 food web pages were visited by trained dietitians to carry out a systematic search of ingredient information from the different food categories. Overall, 1,489 products were analyzed, of which 815 (55.5%) contained at least one NCS, being this proportion particularly high, compared to other countries. 67.1% of the dairy products, 31.5% of the cereal products, 49% of the processed fruits, 74.3% of the non-alcoholic beverages, and 46.2% of sweets and other desserts contained NCS. Considering the food categories more specifically oriented to children, NCSs were present in 98.8% of powder juices, 98.3% of the flavored milks, 91.2% of jellies, and 79% of the dairy desserts. Sucralose and steviol glycosides were the most widely used NCSs, these sweeteners being present, alone or mixed with other, in 73.5 and 39.7% of the NCS-containing products, respectively, while the use of saccharin and cyclamate was low. In addition, 80 tabletop NCSs were available in the local market, 91.2% of them being sucralose and steviol glycosides (alone or combined). The high number of food products containing steviol glycosides makes very plausible that the daily consumption of this NCS in the pediatric populations could exceed its acceptable daily intake (ADI). The fact that there are no NCS-free foods alternatives for certain food categories, especially for children, is worrying.

## Introduction

Chile ranks second in the OECD (Organization for Economic Co-operation and Development) list of countries with higher obesity rates, with a prevalence of 13.1% in 6–7 years school children and 30.5 and 38.8% in adult men and women (from 20 to 49 years), respectively (1, 2). This is an important finding considering that high body mass index and diet-associated risk factors are currently considered as the leading cause of premature death and disability in the world, including in children (3–5). About one-third of the energy consumed by the Chilean population comes from ultra-processed foods, i.e., more than in other Latin-American countries (6). Furthermore, Chileans recently became the greatest consumers of sweetened beverages worldwide (7). With the aim to tackle the constant increase in obesity and non-communicable diseases and fight the growing sales of sugar-sweetened beverages and junk foods, the Chilean government enacted a new Law of Food Labeling and Advertising (Law 20.606) in 2012, which was finally implemented in June 2016 (8, 9). The aim of the law was to restrict the marketing, advertising and sale of non-healthy foodstuffs focused to children and, on the other hand, to specify limits for the content of critical nutrients (sodium, sugars, and/or saturated fats) and total energy in processed, liquid and solid, food products. Consequently, those exceeding the limits for one or more critical nutrient must exhibit a front-of-package warning label consisting of a black hexagon stating in white letters “High in …,” with the identity of the corresponding critical nutrient. Some food products, therefore, may have up to four of these warning labels. Since the initial application of the Law in 2016, these limits have been gradually lowered, first in 2017, and finally in 2018 (10, 11).

To avoid the application of these warning labels, the food companies have been progressively reformulating their products, eliminating, or decreasing their contents of critical nutrients (12). Regarding sweet foodstuffs, an easy solution is the partial or total substitution of sugars by non-caloric sweeteners (NCSs) (13). Some local reports suggest that the number of NCS-containing foodstuffs, particularly beverages and dairy products, has been increasing in Chile during last years (14, 15); however, additional studies are needed to further document the extent of this issue in Chile.

The consumption, both voluntary and involuntary, of these additives is therefore in sharp increase in the population. The involuntary consumption of NCSs has been illustrated by Sylvetsky et al. (16) who detected sucralose in the urine of 44% of subjects who claimed not to consume this sweetener. These authors reported that the presence of sucralose was not indicated in the labeling of some foodstuffs that contained it. In addition, sucralose has not only been used as food additive but also in some pharmaceutical products (syrups, etc.) and cosmetics (toothpaste). It has also been detected in tap water, being currently considered as a new class of pollutant, as well as acesulfame-K. Both NCSs are resistant to the processes used in treatment plants and, therefore, they can be detected in micromolar concentrations in tap water (17).

From a health point of view, a great number of pre-clinical, clinical, and epidemiological studies suggest a lack of effect of NCSs or, even worse, negative effects. For example, the intake of NCSs can induce dissociation between the sweet taste perceived in the mouth and the deficient caloric brought by the food that contains them, a phenomenon that would condition compensatory increases in appetite, energy intake, and weight gain (18, 19). NCSs could also activate sweet taste receptors (STRs) in enteroendocrine cells, upregulating glucose, and fructose intestinal absorption (20, 21). Additionally, they can also affect the composition of the gut microbiota, resulting in glucose resistance in mice and human volunteers (22–24). Consequently, growing evidence suggests that the chronic intake of NCSs does not produce any significant beneficial effects or, worse, affect negatively the consumer's health, increasing obesity and type 2 diabetes (T2D) risk (25, 26), questioning therefore, their nutritional benefits and their increasing use in foods and beverages, more particularly in the pediatric populations (27–29).

Despite these concerns, the consumption of NCSs is growing at a sustained rate in much of the world including Chile. This phenomenon represents an emerging problem in children who are affected from early ages to amounts of NCSs that can exceed their Acceptable Daily Intake (ADI), without knowing the implications of such exposure at the metabolic level (28–32). In Chile, this problem can be considered as an undesirable “throwback” of the new Regulation. Eight NCSs are currently authorized according to the Sanitary Regulation for Food Products: acesulfame K (E-950), aspartame (E-951), cyclamate (and its sodium, potassium and calcium salts) (E-952), saccharin (sodium, potassium and calcium salts) (E-954), sucralose (E-955), alitame (E-956), steviol glycosides (E-960), and neotame (E-961) (33). The Chilean Regulation states that food labeling must indicate the eventual presence of these additives, and their amount per serving and per 100 g or 100 ml of the product ready for consumption. In addition, it must also specify the ADI in mg/kg body weight for each NCS present in the foodstuff, accordingly to the FAO/WHO recommendations.

Based on this background, the aim of this study was to identify the NCS-containing products from different food/beverage categories currently available in the Chilean market and to compare these results with those from other countries.

## Materials and Methods

### Data Source and Sampling

The current study was carried out in Santiago, capital of Chile, that gathers around two-fifths of the whole Chilean population. Eighty-eight percent of food sales in Chile are concentrated in four supermarket chains, led by Walmart Chile with more than 300 stores along the country, followed by CENCOSUD, SMU, and Falabella. A purposeful sampling was used to select nineteen supermarkets belonging to these four chains (Walmart Chile, *n* = 7; CENCOSUD, *n* = 7; Falabella (Tottus), *n* = 4; SMU, *n* = 1). These supermarkets were located in areas of high (Vitacura, Las Condes, and Providencia), medium (La Florida), and medium/low (Puente Alto, Independencia) socio-economic status in Santiago.

### Data Collection

All the supermarkets were visited by fieldworkers between December 2018 and October 2019. The fieldworkers participating in the study were registered dietitians and 3rd-year students from the School of Nutrition and Dietetics at the University of Chile with training in food composition and labeling. Data collection was oriented to the groups of foods whose composition may have been changed to incorporate NCSs in replacement of sucrose, i.e., dairy products, cereal products, processed fruits, non-alcoholic beverages, and sweets and other desserts. Data was also obtained for the different tabletop NCSs available in the supermarkets. The information collected from the food and beverage products included the ingredient list and the nutrition facts label, more particularly the content of caloric and non-caloric sweeteners. Data also include the identity of the NCS(s) present in the products, its concentrations per serving size, and the brand of the product. To facilitate data collection, photos of the packaged food and beverage products were taken and posteriorly analyzed. The nutritional information of the products was also confirmed by accessing the web pages of the supermarkets and/or that of the corresponding food companies. The five food groups previously described were sub-classified in 22 categories, as shown in Table S1. Data was transcribed into an Excel (Microsoft Corporation) spreadsheet for analysis. Information from data entry included the name of the product, the food group, and category to which the product belonged, the brand, and the nutrition facts label per serving and per 100 g.

The proportions of NCS-containing products in each food group were compared with these recently reported in Brazil, Mexico, United States, Spain, Australia, and New-Zealand (34–37).

### Statistical Analyses

All the data were checked for normality using the Shapiro–Wilk test using IBM SPSS Statistics 22.0. The information corresponding to the NCS concentrations was organized according to the food category and presented as median and interquartile range. The presence and frequency of use of the different NCSs were analyzed globally and within each food and beverage subgroup. Subsequently, the individual or combined use of each NCS (with other NCS or added sugars) was assessed in each food group.

## Results

**Figure 1:**
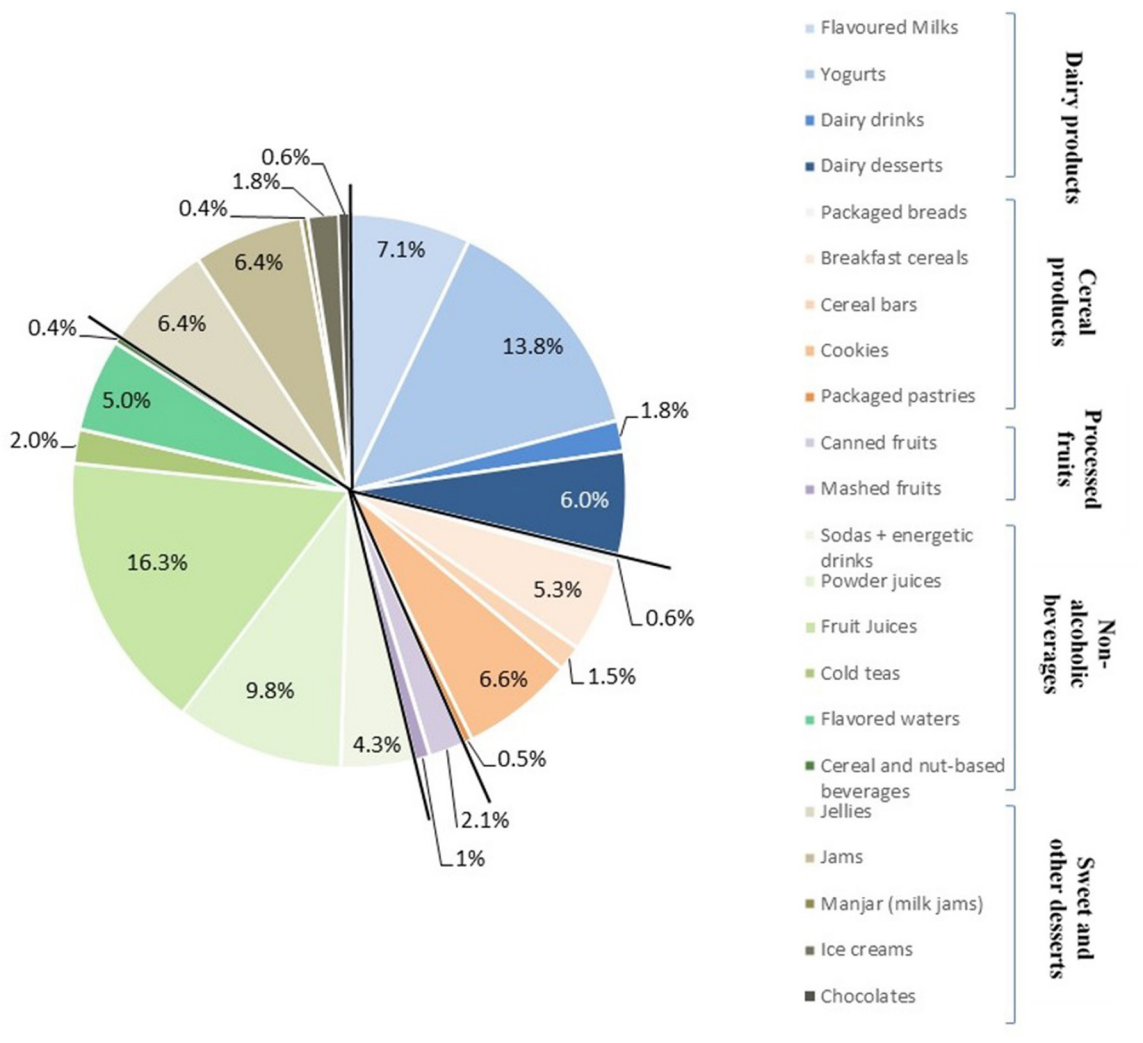
NCS-containing food products by group/category in percent of the total products with NCSs (*n* = 815).

A database compiling the content of NCSs and sugars in 1,489 foods and beverages commercialized in the Chilean market was built, allowing NCS identification and the eventual presence of added sugars. Of all the analyzed products, 815 (55.5%) contained at least one NCS: 38.2% of these NCS-containing products were non-alcoholic beverages, 28.8% dairy products, 15.6% sweets, and other desserts, 14.5% cereal products, and 2.9% processed fruits. Figure 1 shows these proportions by food categories; the five most important were fruit juices (16.3%), yogurts (13.8%), powder juices (9.8%), flavored milks (7.1%), and cookies (6.6%), that represented more than 50% of the total of the NCS-containing products. Table 1 describes the proportion of NCS-containing products by food categories. Notably, 98.3% of the flavored milk, 79% of the dairy desserts, 59.2% of yogurts, and 39.5% of the dairy drinks contained at least 1 NCS so that globally, 67.1% of all the dairy products analyzed contained NCSs. Regarding the cereal products, NCSs were found in 40.9% of the cookies, 39.4% of the breakfast cereals, 23.5% of packaged pastries, 21.8% of the cereal bars, and 8.1% of the packaged breads, i.e., 31.5% of all the cereal products. With respect to the processed fruits, 56.7% of the canned fruits and 36.8% of the mashed fruits have NCSs, i.e., 49% of all the processed fruits. Concerning the non-alcoholic beverages, the presence of NCSs was reported in 100% of the flavored waters, 98.8% of the powder juices, 79% of the iced tea, 72.9% of the soda and energy drinks, and 65.8% of the fruit juices. Together, 74.3% of the non-alcoholic beverages contained NCSs. Finally, in the sweet and other desserts group, 91.2% of the jellies, 63.4% of jams, 23.4% of the ice-creams, 23.1% of milk jams, and 8.5% of chocolates contained NCSs, i.e., 46.2% of this food group.

**Table 1:** Quantity of products with or without NCSs classified by food category.

**Group/subgroup of products**	**Products**	**Products**	**Total**
	**with NCS**	**without NCS**	
**Dairy products**			
Flavored milks	58 (98.3%)	1	59
Yogurts	113 (59.2%)	78	191
Dairy drinks	15 (39.5%)	23	38
Dairy desserts	49 (79.0%)	13	62
(flans rice and semolina milk pudding)			
Total	235 (67.1%)	115	350
**Cereal products**			
Packaged breads	5 (8.1%)	57	62
Breakfast cereals	43 (39.4%)	66	109
Cereal bars	12 (21.8%)	43	55
Cookies	54 (40.9%)	78	132
Packaged pastries	4 (23.5%)	13	17
Total	118 (31.5%)	257	375
**Processed fruits**			
Canned fruits	17 (56.7%)	13	30
Mashed fruits	7 (36.8%)	12	19
Total	24 (49.0%)	25	49
**Non-alcoholic beverages**			
Sodas + energetic drinks	35 (72.9%)	13	48
Powder juices	81 (98.8%)	1	82
Fruit juices	132 (65.7%)	69	201
Cold tea	16 (79.0%)	3	19
Flavored water	44 (100%)	0	44
Cereal, soy, or/and nut-based beverages	3 (11.5%)	23	26
Total	311 (74.3%)	109	420
**Sweet and other desserts**			
Jellies	52 (91.2%)	5	57
Jams	52 (63.4%)	30	82
Milk jam (manjar)	3 (23.1%)	10	13
Ice creams	15 (23.4%)	49	64
Chocolate	5 (8.5%)	54	59
Total	127 (46.2%)	148	275
Total all products	815 (55.5%)	644	1,489

Regarding the number of NCSs present in these products, 42% contained only one NCS, 48.1% had two NCSs, 8.6% three NCSs, and only 1.4% had four NCSs. Table S2 describes, for each category of products the different combinations of NCS they contained. Sucralose was clearly predominant, alone or in combination with other(s) NCS(s) in 19.9 and 53.6%. of the products, respectively. Steviol glycosides were also widely used, being present alone in 7.9% of the products and combined with others NCSs in 31.8%. Aspartame alone was rarely used, only in four products (0.5%), but was more present combined with others NCS (17.1% of the foods). Acesulfame-K alone was only used in four products while its combination with other NCSs was found in 214 foods (26.3%). Saccharin and cyclamate were not frequently used, being present in only 9 (1.1%) and 11 (1.3%) products, respectively, mixed with other NCSs. Alitame and neotame were not detected in any products analyzed in this study.

Table S2 also shows that sucralose and steviol glycosides alone, or less frequently in combination, were exclusively used in bakery products and processed fruit foods. Both NCSs were also predominant in dairy foods while acesulfame-K, aspartame, and cyclamate were present in only 2.8% of these foodstuffs. They were also frequently used in sweets and desserts, being present, alone or combined in 80.3% of the foods. The situation was much more diverse in non-alcoholic beverages. In these categories, sucralose and steviol glycosides were only present in 32.8% and food while acesulfame-K and aspartame were more frequently used. In all these foodstuffs, saccharine and cyclamate were poorly used.

In addition, the concentrations of NCS per 100 g of products that exclusively contained NCSs (i.e., without caloric sweeteners) in the different food categories are shown in Table S3. In general, higher concentrations of sucralose were present in breakfast cereals and canned fruits, while higher concentrations of steviol glycosides were present in cookies, packaged pastries, and canned fruits. High levels of aspartame were found in fruit juice and jellies.

Regarding tabletop NCSs, 80 products were found; 35 in liquid form and 45 in solid form (powder and tablets), as indicated in Table 2. Thirty-five (43.8%) contained sucralose, 15 (18.8%) steviol glycosides, and 23 (28.8%) a combination of sucralose with steviol glycosides. Saccharin and combinations of sucralose with other NCSs were present in less than 10% of tabletop NCSs. Furthermore, 17 of these 80 products also contained other ingredients: tagatose (in 6), agave syrup (in 4), thaumatin (in 2), probiotics (in 2), and fructose (in 1). Additionally, one product contained yacon syrup (an inulin-rich Andean plant) and another, maqui extract (*Aristotelia chilensis*, a polyphenol-rich Chilean berry).

**Table 2:** Tabletop NCSs available in the Chilean market.

**NCS**	**Liquid form**	**Solid form**	**Total**
Saccharin	0	1	1
Steviol glycosides	5	10	15
Sucralose	12	23	35
Saccharin + sucralose	3	0	3
Steviol + sucralose	13	10	23
Sucralose + acesulfame-k	1	0	1
Saccharin + cyclamate	1	0	1
Sucralose + acesufame-k + steviol	0	1	1
Total	35	45	80

Finally, Table 3 summarized the results from similar studies carried out in other countries from Latin America [Brazil (34), Mexico (35)], North America [USA (36)], Europe [Spain (37)], and Oceania [New Zealand and Australia (35)]. Chile shows a much higher proportion of NCS-containing foodstuffs, globally as well as in each considered food group. The second country with higher proportion of NCS-containing foods was Brazil with 24.4%, i.e., less than half what is reported in Chile. The difference between Chile and the other countries was more important when considering dairy products and processed fruits. NCS-containing foods and beverages were rather infrequent (<3%) in Australia and New-Zealand.

**Table 3:** Percentages of products with NCSs in every food group in Chile compared with other countries.

**Food groups**	**Chile**	**Brazil**	**Mexico**	**U.S.A**.	**Spain**	**New Zealand**	**Australia**
	(*n* = 1,489)^*^	(*n* = 1,987)^*^ (34)	(*n* = 7,426)^*^ (35)	(*n* = 28,242)^*^ (36)	(*n* = 763)^*^ (37)	(*n* = 5,325)^*^ (35)	(*n* = 5,152)^*^ (35)
Dairy products	67.1%	14%	25.2%	20.5%	12%	0.1%	0.12%
Cereal products	31.5%	18.2%	5.3%	10.6%	5%	0.4%	0.2%
Processed fruits	49%	10%	NR	3.4%	NR	NR	NR
Non-alcoholic beverages	74.6%	42.4%	40.8%	23.9%	NR	8.7%	2.3%
Sweets and other desserts	46.2%	24.4%	4.9%	14.8%	7.7%	2.8%	0
Total	55.5%	24.4%	19.5%	14.8%	7.7%	2.1%	0.6%

* *Total number of products analyzed in the study. NR, not reported*.

## Discussion

Three years ago, a new Law of Food Labeling and Advertising was implemented in Chile to restrict the sale of unhealthy food for children and specify limits for critical nutrients and energy in food (8–13). Food products overpassing these limits must exhibit a front-of-package warning label advertising the consumer on the high level of critical nutrient they contain. To avoid the presence of these warning labels on their products, food companies have been reformulating the composition of their products, more particularly those “High in sugars” in which sucrose was replaced, partially or totally, by NCSs (14, 15). NCSs are food additives generally recognized as safe (GRAS) by the FDA in U.S.A. and EFSA in the E.U., that are also used in some medicines and health-care products including toothpaste. Their sweetening power may be considerably greater than that of sucrose (100–200 for acesulfame-K and aspartame, 600 for sucralose, until 7,000–13,000 for neotame) and they are not or little metabolized in the organism. They are therefore added in small amounts to foods and beverages in replacement of sucrose, reducing their caloric density.

Accordingly, they have been increasingly used in the world for the prevention and management of obesity and type-2 diabetes (30–32, 38). For example, the recent Ibero-American consensus statement (39) recommends that “foods and beverages with NCS could be included in dietary guidelines as alternative options to products sweetened with free sugars.” However, this document gives little importance to the fact that, during the last decade, a great number of studies, both in animals and humans have questioned their nutritional benefits, suggesting an absence of effect or, in some cases, negative effects (18–29). Noteworthily, EFSA rejected the requests of Health Claims relative to the effect of NCSs on weight control and the maintenance of normal glycemia (40) and the Pan American Health Organization, in its recently published “Nutrient Profile Model,” included NCSs as other critical nutrients with the aim to discourage the consumption of processed and ultra-processed food products and to promote that of unprocessed and minimally processed foods (41). This is therefore a very conflictive problem and more studies are urgently needed to elucidate whether NCSs are useful in the management of obesity and its complications, more particularly in the pediatric population (28, 29). Until so, it is important that the consumers can make their choice between sugar- or NCS-sweetened foods and beverages.

The present study was carried out in this context, to identify the NCS-containing products from different food/beverage groups and categories currently available in different supermarkets in Santiago, Chile. We analyzed the food labeling of 1,489 foodstuffs belonging to five main groups of processed foods/beverages, observing that 55.5% of them contained at least one NCS. In four categories (flavored waters, powder juices, flavored milks, and jellies) more than 90% of the corresponding products contained NCS, three of them (powder juices, jellies, and flavored milks) being more specifically oriented to children. Furthermore, more than 50% of yogurts, dairy desserts, canned fruits, sodas, fruit juices, cold teas, and jams contained NCS. Again, all these products (except cold teas) are highly consumed by children. In addition, the Chilean market also exhibits a huge amount (80) of tabletop NCSs, most of them (96.6%) containing sucralose, steviol glycosides, and combinations sucralose/steviol glycosides.

When the representativeness of the food products assessed in the study was compared to the data obtained from the last Chilean household budget survey, these indicate that the food budget represents 18.7% of the total monthly expenses, 18.3% of them being used to purchase of bread and cereal products, about 18% for non-alcoholic beverages 11.3% for dairy products, and 4.3% for sweet products (42). Consequently, 51.9% of the food expenses are destined to buy processed foods that eventually might contain NCSs.

On the other hand, we also compared our results with those from similar studies carried out in other countries (34–37). Chile was clearly the country with the highest proportion of NCS-containing food products (55.5%), Brazil being the second with 24.4%, i.e., less than half what is reported in Chile. This difference was even higher when considering Dairy products or Processed fruits.

The proportion of NCS-containing foods and beverages is therefore very high in Chile, increasing the probability of involuntary intake of NCSs and reducing the possibility of choosing foods without these additives for the consumers. More particularly, a high proportion of food products specifically oriented to children contained NCSs. These results are important as they suggest that Chilean children could ingest higher amounts of NCSs than in other countries, possibly exceeding their ADI. This is more plausible in the case of steviol glycosides whose ADI is low, 4 mg/kg of body weight (considering its steviol content), compared with the other NCSs. For example, it is plausible that a 7 year old Chilean child can consume during the day a portion of chocolate flavored milk (200 ml) and corn flakes (30 g), an applesauce (100 g), a cereal bar, a flavored jelly (100 g), a yogurt (125 g), and 2.5 portions of fruit juice (0.5 L) that could bring, according our database, 24, 21.6, 20, 13, 56, 37.5, and 160 mg of steviol glycosides, respectively, i.e., 332 mg in total or a corresponding amount of steviol of 132.8 mg approximately. If this child is weighing 22.5 kg, therefore, he will be ingesting 5.9 mg of steviol per kg of body weight, i.e., 1.47 times the ADI for this NCS. Notably, steviol glycoside appeared in the Chilean market in 2009, and a study evaluating the daily intake of NCSs in 281 Chilean children and published in 2011 did not report any consumption of this NCS in this population (43). Our results, therefore, show that eight years later, the situation has dramatically changed since 21.8% of all the food products evaluated in our study contained steviol glycosides (39.8% of the NCS-containing foods and beverages). In their study in Chilean children, Durán et al. (43) reported that the intake of aspartame, acesulfame-K, and sucralose reached 11.8, 11.3, and 18% of the respective ADI of these NCSs. From our results, sucralose is currently the most used NCS in Chile and it is probable that this percentage has increased since 2011. An important aspect is that ADI is estimated for each NCS, independently from each other, and it is totally unknown how these NCS interact in the human body and what are the consequences of these interactions on human health, more particularly in the pediatric population (25–27).

Finally, our study also reports the median concentrations of NCS in the different food categories. This was possible because the Chilean Regulation states that food labeling must indicate the amount of these additives per serving and for 100 g or 100 ml of the product ready for consumption. This is notable as most of the countries in the world do not exhibit this data on their food labeling.

This study is the first carried out after the second and third step of implementation of the Law 20.606 of Food Labeling and Advertising aiming to reduce the presence of critical nutrients and energy in processed food products. Its main limitation is that it was carried out only in Santiago and the situation may differ in the most rural areas and other cities of the country. Some NCS-containing foodstuffs available in Santiago may not be available in some regions and conversely, some products may be elaborated specifically in some regions without reaching Santiago. However, this potential bias is probably minimized because the supermarket chains used in the study are present in all the Chilean cities where they represent 88% of the whole food sales. On the other hand, our study did not include processed foods imported from Asia and some neighboring countries, which frequently do not exhibit adequate food labeling. Finally, it must be stated that food formulations and products are very changeable and therefore, the database elaborated in the current study needs to be regularly updated.

## Conclusions

Our results show that a high proportion of the foods and beverage products available in the different supermarket chains of Santiago contains NCS, compared with other countries. The NCSs most frequently added to food products or available as tabletop NCSs are sucralose and steviol glycosides, while the use of saccharin and cyclamate is rather low. The fact that there are no alternatives of foods without NCS for certain food categories, specially targeted children, is worrying. The high number of food products containing steviol glycosides makes very plausible that the daily consumption of this NCS in the pediatric populations could exceed its ADI. A priority for the future investigations is to determine the health impact of the chronic simultaneous intake of several NCS. Finally, food formulations and products evolve very quickly, according to tastes and fashions. More studies are therefore needed in future to determine the evolution of the NCS-containing foods and beverages. This study represents a starting point for improving the regulation concerning the use of NCSs in food products in Chile.

## Data Availability Statement

The datasets generated for this study are available on request to the corresponding author.

## Author Contributions

The study was designed by VS, SL-A, PC, BC, and MG. KA, VS, JC, AE, SV, GP, and RP were responsible of the identification the NCS-containing products in the different supermarkets and web pages. SL-A, VS, RP, and PC compile the food composition database. VS, MG, and SL-A analyzed the data. MG, VS, SL-A, and KA wrote the manuscript. All the authors revised and approved the manuscript.

## Conflict of Interest

The authors declare that the research was conducted in the absence of any commercial or financial relationships that could be construed as a potential conflict of interest.
