# Supplementary material for: Overuse of Non-caloric Sweeteners in Foods and Beverages in Chile: A Threat to Consumers' Free Choice?
Source: Front Nutr. 2020 Jun 17;7:68. doi: 10.3389/fnut.2020.00068 (PMC7311776; doi:10.3389/fnut.2020.00068)
Supplement: Supplementary file 1 [file Data_Sheet_1.docx]

Supplementary Material

Table S1. Groups and categories of foods evaluated for the presence of non-caloric sweeteners (NCSs).

| Groups | Categories | Number of brands |
| --- | --- | --- |
| Dairy products | Flavoured Milk  Yogurts  Dairy drinks  Dairy desserts | 17  12  9  9 |
| Cereal products | Packaged breads  Breakfast cereals  Cereal bars  Cookies  Packaged pastries | 10  18  13  9  3 |
| Processed fruits | Canned fruits  Mashed fruits | 9  3 |
| Non-alcoholic beverages | Sodas + energetic drinks  Fruit juices  Powder juices  Cold tea  Flavored water  Cereal-, nut-based beverages | 9  32  6  6  5  13 |
| Sweet and other desserts | Jellies  Jams  Milk jams  Ice creams  Chocolate | 12  14  10  12  26 |

**Table S2.** NCS concentrations in different food categories (mg/100g) Median (Interquartile range).

| **Category of Products** | **Sucralose** | **Saccharin** | **Acesulfame-k** | **Cyclamate** | **Steviol glycoside** | **Aspartame** |
| --- | --- | --- | --- | --- | --- | --- |
| Flavored Milks | 8  (4.5 – 11.5) | - | 6  (6 - 9) | - | 12  (10 - 14) | - |
| Yogurts | 12  (12 - 15) | - | 22  (11.5 - 22.8) | - | 15  (5 - 19) | 22.5 |
| Dairy drinks and substitutes | 15  (14 -171) | - | - | - | 11.9  (11.8 - 11.9) | - |
| Dairy desserts | 8.3  (8 - 11) | - | 9  (9-11.5) | 47 | 13  (13 – 16.6) | - |
| Packaged breads | 2.7 | - | - | - | - | - |
| Breakfast cereals | 32  (28.2 – 45.2) | - | - | - | 33.5  (22.0 – 42.8) | - |
| Cereal bars | 11.6  (10.8 - 16.6) | - | - | - | 1  (0.04 – 2.0) | - |
| Cookies | 16.4  (14.1 – 24.5) | - | - | - | 45  (41 -47) | - |
| Packaged pastries | 11.4 | - | - | - | 48 | - |
| Canned fruits | 28.4  (18.7 -30.0) | - | - | - | 48 | - |
| Mashed fruits | 20  (12 - 20) | - | - | - | 12  (11 - 16) | - |
| Sodas + energy drinks | 17.3  (4.6 – 21.5) | 9 | 11  (9 - 14) | 25 | - | 23  (15.5 - 34.8) |
| Powder juices | 7  (2 – 8) |  | 9  (7 - 15) |  | 15.7  (13.8 – 21.5) | 25  (22.0 – 30.8) |
| Fruit Juices | 8  (4.0 – 15.5) | - | 7  (6 - 8) | - | 11.7  (2.7 – 19.3) | 31  (3.5 – 23.7) |
| Cold teas | 10  (9 - 13) | - | 8  (6 - 9) | - | 10  (3.9 – 10.0) | - |
| Flavored waters | 7.5  (5.3 – 10.5) | - | 2.9  (2.6 - 3) | - | 8.7  (5.2 – 9.0) | 9 |
| Jellies | 20  (14.0 - 24.3) | - | 11  (11 - 12) | - | 40  (19 - 56) | 34 |
| Jams | 20  (13.6 – 30.0) | - | 3.5  (3.4 - 3.6) | - | 22.7  (20 - 29) | - |
| Milk jams | 7  (6 - 10) | - | - | - | 8  (7 - 8) | - |
| Ice creams | 2  (2 -3) | - | - | - | 5  (4 - 5) | - |

**Table S3**. Number of food products with non-caloric sweeteners by food category and type of NCS, alone or combined.

| **Food Category** | **SUC** | **SAC** | **ACE-K** | **CYC** | **ASP** | **SG** | **N°** |
| --- | --- | --- | --- | --- | --- | --- | --- |
| Flavored Milks | ⚫  ⚫  ⚫ |  | ⚫ |  |  | ⚫ | 25  26  7 |
| Yogurts | ⚫  ⚫  ⚫  ⚫ |  | ⚫ |  | ⚫ | ⚫  ⚫  ⚫ | 47  9  36  14  7 |
| Dairy drinks | ⚫ |  |  |  |  |  | 15 |
| Dairy desserts | ⚫  ⚫  ⚫ |  | ⚫  ⚫  ⚫ | ⚫ |  | ⚫  ⚫ | 22  4  14  3  4  2 |
| Packaged Breads | ⚫ |  |  |  |  |  | 5 |
| Breakfast cereals | ⚫  ⚫ |  |  |  |  | ⚫  ⚫ | 14  6  23 |
| Cereal bars | ⚫  ⚫ |  |  |  |  | ⚫ | 8  4 |
| Cookies | ⚫  ⚫ |  |  |  |  | ⚫  ⚫ | 34  11  9 |
| Packaged pastries | ⚫ |  |  |  |  | ⚫ | 2  2 |
| Canned fruits | ⚫  ⚫ |  |  |  |  | ⚫ | 16  1 |
| Mashed fruits | ⚫  ⚫ |  |  |  |  | ⚫ | 4  3 |
|  |  |  |  |  |  |  |  |
| Sodas + energy drinks | ⚫  ⚫ | ⭘ | ⭘  ⚫  ⚫ | ⭘ | ⭘  ⚫  ⚫ |  | 1  4  16  12  2 |
| Powder juices | ⚫  ⚫  ⚫  ⚫  ⭘ |  | ⚫  ⚫  ⚫  ⚫  ⭘ |  | ⚫  ⚫  ⭘ | ⚫  ⚫  ⭘ | 7  2  30  3  34  6 |
| Fruit juices | ⚫  ⚫  ⚫  ⚫  ⚫ |  | ⚫  ⚫  ⚫ |  | ⚫  ⚫  ⚫  ⚫ | ⚫  ⚫ | 36  19  38  8  2  2  13  48 |
| Cold tea | ⚫  ⚫  ⚫ |  | ⚫  ⚫ |  | ⚫ | ⚫  ⚫ | 4  2  4  5  1 |
| Flavored water | ⚫  ⚫  ⚫  ⚫ |  | ⚫  ⚫ |  | ⚫ | ⚫ | 4  21  18  1 |
| Cereal, nut-based beverages | ⚫  ⚫ |  | ⚫ |  |  | ⚫  ⚫ | 1  1  1 |
| Jellies | ⚫  ⚫  ⚫ | ⚫ | ⚫  ⚫ | ⚫ | ⚫  ⚫ | ⚫ | 11  20  11  5  5 |
| Jams | ⚫  ⚫  ⚫ |  | ⚫ |  |  | ⚫  ⚫ | 16  31  4  1 |
| Milk jams | ⚫  ⚫ |  |  |  |  | ⚫ | 1  2 |
| Ice creams | ⚫  ⚫ |  |  |  |  | ⚫  ⚫ | 5  2  8 |
| Chocolates | ⚫ |  |  |  |  | ⚫ | 2  3 |

⚫ SUC ⚫ SUC + SG ⚫ SAC + CYC ⚫ SUC + ASP + SG

⚫ SG ⚫ SUC + ACE –K ⚫ SUC + ASP ⚫ SUC + ACE-K + SG

⚫ ACE-K ⚫ ACE-K + CYC ⚫ SUC + ACE K + ASP

⚫ ASP ⚫ ACE-K + ASP ⭘ >3 NCSs

SUC = Sucralose; SG = Steviol glycosides; ACE-K = Acesulfame-K;

ASP = Aspartame; CYC = Cyclamate; SAC = Saccharine.
